# Recent Advances in Dye-Sensitized Solar Cells

**DOI:** 10.3390/molecules26092461

**Published:** 2021-04-23

**Authors:** Claudia Dragonetti, Alessia Colombo

**Affiliations:** Department of Chemistry, Università degli Studi di Milano, Via Golgi 19, 20133 Milano, Italy

Dye-sensitized solar cells (DSSCs) are an effective alternative for delivering clean energy from the sun compared to the most widely deployed technologies based upon semiconductor photovoltaics. DSSCs can convert solar light into electricity thanks to some key elements: conducting glass covered by a layer of a wide-bandgap semiconductor (typically TiO_2_ nanoparticles) functionalized with compounds able to efficiently adsorb the solar light. The other, but not less important, components are the electrolyte (a redox couple able to transport electrons and to regenerate the dye) and the counter electrode (conductive glass coated with a thin layer of platinum).

The present Special Issue is a collection of 2 reviews and 12 articles covering some of the most recent advances in DSCC components both from experimental and theoretical points of view.

In the first review, Zhou et al. [1] summarized the current progress on dye aggregation as a potential approach to improve device performance. Several methods for regulating dye aggregation in liquid and solid-state DSSCs were presented, and their advantages and disadvantages were analyzed. In addition, the authors showed the importance of the theoretical calculation as a powerful tool to predict the dye aggregation pattern. 

Although redox mediators are essential components of the solar cell, they have received much less research effort than dyes. The use of copper complexes as alternative redox mediators is the focus of the second review. Fagnani et al. [2] reported the most recent (from 2016 to the present) developments in the use of Cu-based redox couples as a low-cost alternative to the widely used I^-^/I_3_^-^ redox mediator.

Copper complexes also found application as dyes in DSSCs; Constable et al. [3] reported the syntheses and characterization of four homoleptic copper (I) complexes and studied their performances in the cell. They underlined the importance of the ligand design, confirming that the introduction of alkynyl spacers between donor and acceptor moieties is not beneficial in terms of efficiency.

Heteroleptic copper (I) complexes as dyes were investigated from a theoretical point of view by Wei et al. [4] using density functional theory (DFT) and time-dependent DFT (TD-DFT) methods. The authors focalized their attention on the nature of the anchoring groups and how the type of anchor influences the electronic structure, the absorption properties and the intramolecular and interfacial electronic transfer.

Phthalocyanines, thanks to their high photochemical and electrochemical stabilities and their strong light-harvesting capability due to a very intense absorption band in the red-NIR region, are another promising class of photosensitizers. Makseed et al. [5] reported the synthesis, characterization and in-cell performances of a new asymmetric push–pull zinc phthalocyanine.

Organic dyes have been demonstrated to be a valid alternative to metal complexes due to their simple synthesis, ease of large-scale production, low cost, flexible molecular design and high molar extinction coefficients.

In the field of metal-free dyes, Koivisto et al. [6] explored the in-cell performances of a bio-inspired family of bichromic–bipodal organic dyes. These compounds show a D–π–D–A architecture where two triphenylamine units act as donors while two cyanoacetic acid units act as acceptors.

Hoff and co-workers [7] focused their attention on organic dyes containing the phenothiazine scaffold; they synthesized and characterized new 3,8-substituted phenothiazine derivatives, instead of the common 3,7-substitution pattern, in order to investigate the effect of auxiliary donor groups on the performances of this novel dye family.

The most commonly used organic dyes are generally synthesized via sequential transformation of building blocks by cross-coupling reaction catalyzed by transition metals, a strategy that often requires the preparation of toxic organometallic units (for example, boronic acid/esters and aryl stannanes). Gautun et al. [8] displayed the potential of direct C–H arylation, a recent C–C bond-forming method extensively employed in the polymer syntheses, in the preparation of five new 3,6-dithienyl diketopyrrole dyes.

Theoretical calculations are a valuable resource in the understanding of the performances of metal-free organic dyes, which allows designing new photosensitizers in a search for the best efficiencies. Baldenebro-Lopez and co-workers [9,10] studied the electronic properties of new and already reported carbazole-based and triphenylamine-based dyes for DSSCs.

Combining experimental and computational studies, Youm et al. [11] were able to establish some structure–property correlations and to give a microscopic explanation of the experimental behavior observed in devices that use chrysanthemin as a photosensitizer.

In addition to the dye and the redox shuttle, another very important DSSC component is the mesoporous TiO_2_ layer; it provides both an extended surface area for the adsorption of the dye and a sufficient immersion of the hole-carrying liquid electrolytes, increasing the light-harvesting capability and dye regeneration efficiency.

Density functional theory with Perdew–Burke–Ernzerhof functional approach was proposed by Maluta et al. [12] to explore the optical and electronic properties of some modeled TiO_2_ brookite clusters, namely (TiO_2_)_n =5,8,68._

In order to control and improve the solar cell performance and efficiency, it is also helpful to model the charge transfer during the sensitization process. In this context, Maldon and Thamwattana [13] proposed a new mathematical model based on fractional diffusion equation, taking into account the random walk network of titanium dioxide, for evaluating the DSSC efficiency.

It is known that by playing with the titanium dioxide conduction band position it is possible to increase the solar cell efficiency; in particular, its negative shift can increase the V_oc_ value as it is directly proportional to the difference in energy between the TiO_2_ conduction band and the redox potential of the redox couple. For this purpose, Han and co-workers [14] reported the use of surface-modified photoanodes obtained by simple soaking of TiO_2_ films in an aqueous solution of a strong base such as Na_2_S.

In conclusion, a great amount of work has already been done on the different components of the solar cell, but more will still need to be done in the future to make these promising devices a competitive technology.

The guest editors would like to thank all the colleagues who contributed to this Special Issue by presenting their recent works. We also thank all the MDPI staff for their important and constant support.
